# Short-term prognostic value of clinical data in hospitalized patients with intermediate-risk acute pulmonary embolism

**DOI:** 10.1186/s12872-022-02783-7

**Published:** 2022-07-28

**Authors:** Jichun Liu, Yuanyuan Liu, Feilong Zhang, Cong Fu, Yang Ling, Ping Fang, Xiangrong Xie, Xianghai Wang, Hao Yang, Youquan Wei, Jinfeng Wang

**Affiliations:** Department of Cardiology, The First Affiliated Hospital of Wannan Medical College (Yijishan Hospital of Wannan Medical College), Wuhu, 241001 Anhui People’s Republic of China

**Keywords:** Acute pulmonary embolism, Arterial oxygen saturation, Right to left ventricular short-axis ratio, Syncope, Negative T waves, White blood cell

## Abstract

**Background:**

Intermediate-risk acute pulmonary embolism (APE) patients are usually defined as hemodynamically stable, comprehending a great therapeutic dilemma. Although anticoagulation therapy is sufficient for most intermediate-risk APE patients, some patients can deteriorate and eventually require a systemic fibrinolytic agent or thrombectomy. Hence, this study aimed to evaluate the predictive value of differences in clinical data for the short-term prognosis of intermediate-risk APE patients.

**Methods:**

A retrospective cohort of 74 intermediate-risk APE patients confirmed by computed tomography pulmonary angiography was analyzed in the present study. Adverse clinical event outcomes included PE-related in-hospital deaths, critical systolic blood pressure consistently under 90 mmHg, refractory to volume loading and vasopressor infusion requirements, mechanical ventilation, and cardiopulmonary resuscitation. The APE patients were stratified into two groups: adverse outcome (n = 25) and control (n = 49) groups. Then, the clinical data of the two groups were compared. Receiver operating characteristic (ROC) curves were used to explore the predictive value of white blood cell (WBC) counts and the right to left ventricular short-axis (RV/LV) ratio. Model calibration was assessed using the Hosmer–Lemeshow goodness-of-fit statistic.

**Results:**

The brain natriuretic peptide, WBC count, and the RV/LV ratio were higher in patients with adverse outcomes compared to controls. The APE patients with adverse outcomes presented significantly higher rates of syncope, Negative T waves (NTW) in V1–V3, intermediate-high risk, thrombolytic therapy, and low arterial oxygen saturation (SaO_2_) compared to controls. In the multivariate logistic regression analysis, the SaO_2_ < 90%, [odds ratio (OR) 5.343, 95% confidence interval (CI) 1.241–23.008; *p* = 0.024], RV/LV ratio (OR 7.429, 95% CI 1.145–48.209; *p* = 0.036), Syncope (OR 12.309, 95% CI 1.702–89.032; *p* = 0.013), NTW in V1–V3 (OR 5.617, 95% CI 1.228–25.683; *p* = 0.026), and WBC count (OR 1.212, 95% CI 1.035–1.419; *p* = 0.017) were independent predictors of in-hospital adverse outcomes among APE patients. The ROC curve analysis indicated that the RV/LV ratio can be used to predict adverse outcomes (AUC = 0.748, *p* < 0.01) and calibration (Hosmer–Lemeshow goodness of fit test, *p* = 0.070). Moreover, an RV/LV ratio > 1.165 was predictive of adverse outcomes with sensitivity and specificity of 88.00 and 59.20%, respectively. The WBC counts were also able to predict adverse outcomes (AUC = 0.752, *p* < 0.01) and calibration (Hosmer–Lemeshow goodness of fit test, *p* = 0.251). A WBC count > 9.05 was predictive of adverse outcomes with sensitivity and specificity of 68.00 and 73.50%, respectively.

**Conclusion:**

Overall, a SaO_2_ < 90%, RV/LV ratio, Syncope, NTW in V1–V3, and WBC counts could independently predict adverse outcomes in hospitalized intermediate-risk APE patients.

## Introduction

Acute pulmonary embolism (APE) is a clinical and pathophysiological syndrome caused by the obstruction of pulmonary circulation. This obstruction is promoted by the blockage of the trunk and the branches of the pulmonary artery by endogenous or exogenous emboli. APE is the third most common acute vascular disease worldwide with a mortality rate behind only myocardial infarction and stroke. The European Union epidemiological model estimates that about 34% of APE patients suddenly die within hours before treatment even begins or becomes effective.

Risk stratification is considered a critical step to select APE treatment strategies during short-term prognosis [1]. Usually, the PE severity index (PESI) and simplified PESI scores are used to predict the 30-day mortality of APE patients [2]. Two clinical prognostic scores are used to identify patients with a low risk of death within 30 days of treatment. However, clinical treatments should be based on broader results, such as cardiopulmonary resuscitation, mechanical ventilation, as well as vasopressor infusion requirement, not only on PE-related deaths [3]. Hence, clinicians should pay special attention and choose reperfusion treatment for PE patients with hemodynamic instability and high risk. Partial or complete outpatient treatments might be used for low-risk APE patients based on their early complications [1]. In the present study, we focused on intermediate-risk APE patients, usually defined as hemodynamically stable, comprehending a great therapeutic dilemma. According to current guidelines, these patients should be rigorously monitored for any sign of hemodynamic instability, since some can deteriorate and eventually require reperfusion therapy (systemic fibrinolytic agents or thrombectomy) [4]. Therefore, better risk stratification strategies are required. Herein, we evaluated the predictive values of the differences in clinical data for the short-term prognosis of intermediate-risk APE patients.

### Patients and methods

#### Clinical data collection

A total of 74 APE patients treated at the First Affiliated Hospital of Wannan Medical College from January 2017 to December 2021 were enrolled in the present study. The inclusion criteria were defined according to the 2019 ESC/ERS Guidelines for the Diagnosis and Management of Acute Pulmonary Embolism [1]. All patients were verified by computed tomography pulmonary angiography (CTPA) and underwent ECG examination, Venous blood sampling, and cardiac ultrasound within 6 h before and after PE diagnosis. Other clinical data, such as gender, age, disease history, onset signs, D-dimer, Serum Creatinine, BNP, and troponin I were also recorded in detail. Patients with chronic thromboembolic pulmonary hypertension, acute coronary syndrome, valvular heart disease, cardiomyopathy, myocarditis, pulmonary artery tumor, electrolyte disturbance, clinical history, and incomplete auxiliary examination data were excluded. Patients with echocardiographic signs of right ventricular dysfunction and/or positive biomarkers were classified with intermediate risk according to the 2019 ESC/ERS Guidelines for the Diagnosis and Management of Acute Pulmonary Embolism [1]. All patients diagnosed with pulmonary embolism were initially treated with low molecular weight heparin in a weight-adjusted dose. In-hospital deaths related to PE, hemodynamic instability, vasopressor infusion requirements, mechanical ventilation, and cardiopulmonary resuscitation were considered adverse outcomes. After diagnosis, routine blood samples were collected for blood cell analysis, and brain natriuretic peptide (BNP), troponin I, and arterial blood gas analysis.

### Electrocardiography and CTPA

The paper speed of conventional 12-lead ECG was 25 mm/s, and the standard voltage was 10 mV. ECG signs were analyzed for right ventricular strain considering the following features: (1) sinus tachycardia; (2) S1Q3T3; (3) Complete or incomplete right bundle branch block (RBBB); (4) supraventricular tachycardia; (5) prolonged QTC interval; (6) Negative T waves (NTW) of lead V1–V3; (7) V1 in Qr; and (8) Clock transposition. Sinus tachycardia was defined as sinus heart rate > 100 beats/min. The S1Q3T3 was defined as an S wave in lead I > 1.5 mm, and Q in lead III > 1.5 mm associated with an NTW in lead III [5]. The RBBB and clock transposition were consistent with traditional diagnostic criteria [5, 6]. The V1 in Qr was defined as a Q wave amplitude ≥ 0.2 mV and width < 120 m s[7]. The NTW was defined as negative T wave amplitude ≥ 0.5 mV [8]. The QTC interval prolongation was defined as QTC ≥ 460 ms [9]. Finally, the cardiac axial CT image was used to measure the RV and LV short-axis diameters at their widest point [10, 11].

### Statistical analyses

The SPSS 18.0 statistical software was used to process the data. Data with normal distribution are presented as ${\overline{\text{x}}} \pm {\text{s}}$. Comparisons between two groups were conducted using t-tests. Data with non-normal distribution are presented as M (P25; P75) and were compared using Mann–Whitney *U* rank tests. Fisher’s exact and χ^2^ tests were used to evaluate categorical variables. To identify the markers used to predict and estimate in-hospital adverse outcomes, we conducted a multivariate logistic regression analysis. Then, ROC curves and Hosmer–Lemeshow were used to confirm the value of continuous data to predict adverse outcomes. A *p* < 0.05 was considered statistically significant.

## Results

A total of 49 (66.2%) controls and 25 (33.8%) adverse outcome APE patients were enrolled in this study. The laboratory findings, predisposing factors, and baseline demographic characteristics are presented in Table 1. The brain natriuretic peptide, WBC count, and RV/LV ratio were significantly higher in patients with adverse outcomes compared to control APE patients. The patients with adverse outcomes also presented higher Syncope, NTW in V1–V3, intermediate high risk, and thrombolytic therapy rates. On the other hand, these patients presented lower SaO_2_ compared to controls (Table 2).

**Table1:** Clinical, electrocardiographic, laboratory, and computed tomographic findings

Variables	Control (n = 49)	Adverse outcome (n = 25)	*p*
Age, y, mean	67.98 ± 12.02	61.80 ± 14.94	0.058^b^
Gender, male	29(59.2)	18(72.0)	0.318 ^a^
Prior DVT	5(10.2)	4(16.0)	0.476 ^a^
Previous surgery/trauma	9(18.4)	9(36.0)	0.151 ^a^
Previous tumour	3(6.12)	1(4.0)	0.703^a^
Smoking	9(18.4)	6(24.0)	0.559 ^a^
Previous COPD	6(12.2)	1(4.0)	0.411 ^a^
Hypertension	24(49.0)	13(52.0)	0.806^a^
Diabetes mellitus	6(12.2)	5(20.0)	0.492 ^a^
Previous CAD	9(18.4)	2(8.0)	0.314 ^a^
Bp(systolic) < 100 mmHg	5(10.2)	6(24.0)	0.167^a^
SaO_2_ < 90%	8(16.3)	10(40.0)	0.043 ^a^
Thrombolytic therapy	6(12.2)	14(56.0)	< 0.010 ^a^
Major bleeding requiring transfusion	0(0)	2(8.0)	0.111 ^a^
RV/LV ratio	1.17 ± 0.48	1.51 ± 0.52	0.006^b^
Syncope	4(8.2)	7(28.0)	0.039 ^a^
Intermediate high risk	25(51.0)	20(80.0)	0.023 ^a^
Tachycardia	22(44.9)	16(64.0)	0.145 ^a^
S1Q3T3	10(20.4)	8(32.0)	0.39 ^a^
RBBB	10(20.4)	3(12.0)	0.523 ^a^
Atrial fibrillation or flutter	3(6.12)	1(4.0)	0.703 ^a^
Qr in V1	5(10.2)	2(8.0)	0.759^a^
NTW in V1–V3	8(16.3)	12(48.0)	0.006 ^a^
QTc prolongation	16(32.7)	13(52.0)	0.134 ^a^
Clockwise rotation	4(8.2)	6(24.0)	0.078 ^a^
WBC, × 109/L	7.60(5.85–8.95)	10.30(8.30–13.30)	0.000 ^c^
Haemoglobin, g/L	126.08 ± 19.10	123.48.10 ± 22.52	0.604^b^
BNP, pg/mL	393.25 ± 374.87	648.66 ± 478.70	0.014^b^
D-dimer,ug/mL	4.90(3.35–7.16)	6.08(4.13–17.11)	0.315 ^c^
Troponin I positive	44(89.7)	22(88.0)	0.814 ^a^
Serum Creatinine, umol/L	70.6(62.80–97.90)	66.0(55.70–77.25)	0.177 ^c^
Length of hospital stay, days	11(9–13)	12(9.5–13.5)	0.094 ^c^

*DVT,* deep venous thrombosis,*COPD*,chronic obstructive pulmonary disease,*CAD*,coronary artery disease,*Bp*,Blood pressure, *SaO*_*2*_,arterial oxygen saturation, *BNP*,brain natriuretic peptide, *RV/LV*,right ventricular short-axis to left ventricular shor-taxis, *NTW*,Negative T waves.*WBC*,white blood cell

*p* < 0.05 indicates statistical significance and was shown in bold characters

^a^Chi-squared

^b^independent-samples t test

^c^Mann–Whitney U test

**Table 2:** Univariate and multivariate analysis for in-hospital Adverse outcome in patients with intermediate risk pulmonary embolism

Univariate analysis	*p*	OR (95% CI)	Multivariate analysis	*p*	OR (95% CI)
SaO2 < 90%	0.029	1.135 (1.135–10.283)	SaO_2_ < 90%	0.024	5.343(1.241–23.008)
RV/LV ratio	0.015	4.133 (1.323–12.909)	RV/LV ratio	0.036	7.429 (1.145–48.209)
Syncope	0.031	4.375 (1.140–16.785)	Syncope	0.013	12.309(1.702–89.032)
NTW in V1-V3	0.005	4.731 (1.590–14.080)	NTW in V1-V3	0.026	5.617 (1.228–25.683)
Intermediate high risk	0.019	3.840 (1.242–11.873)			
BNP	0.022	1.001 (1.000–1.003)			
WBC, × 10^9^/L	0.020	1.145 (1.022–1.282)	WBC, × 10^9^/L	0.017	1.212 (1.035–1.419)

*RV/LV,*right ventricular short-axis to left ventricular short-axis, *BNP*,brain natriuretic peptide, *NTW*,Negative T waves, *WBC*,white blood cell, *SaO*_*2*_*,*arterial oxygen saturation

*p* < 0.05 indicates statistical significance and was shown in bold characters

**Fig. 1:**
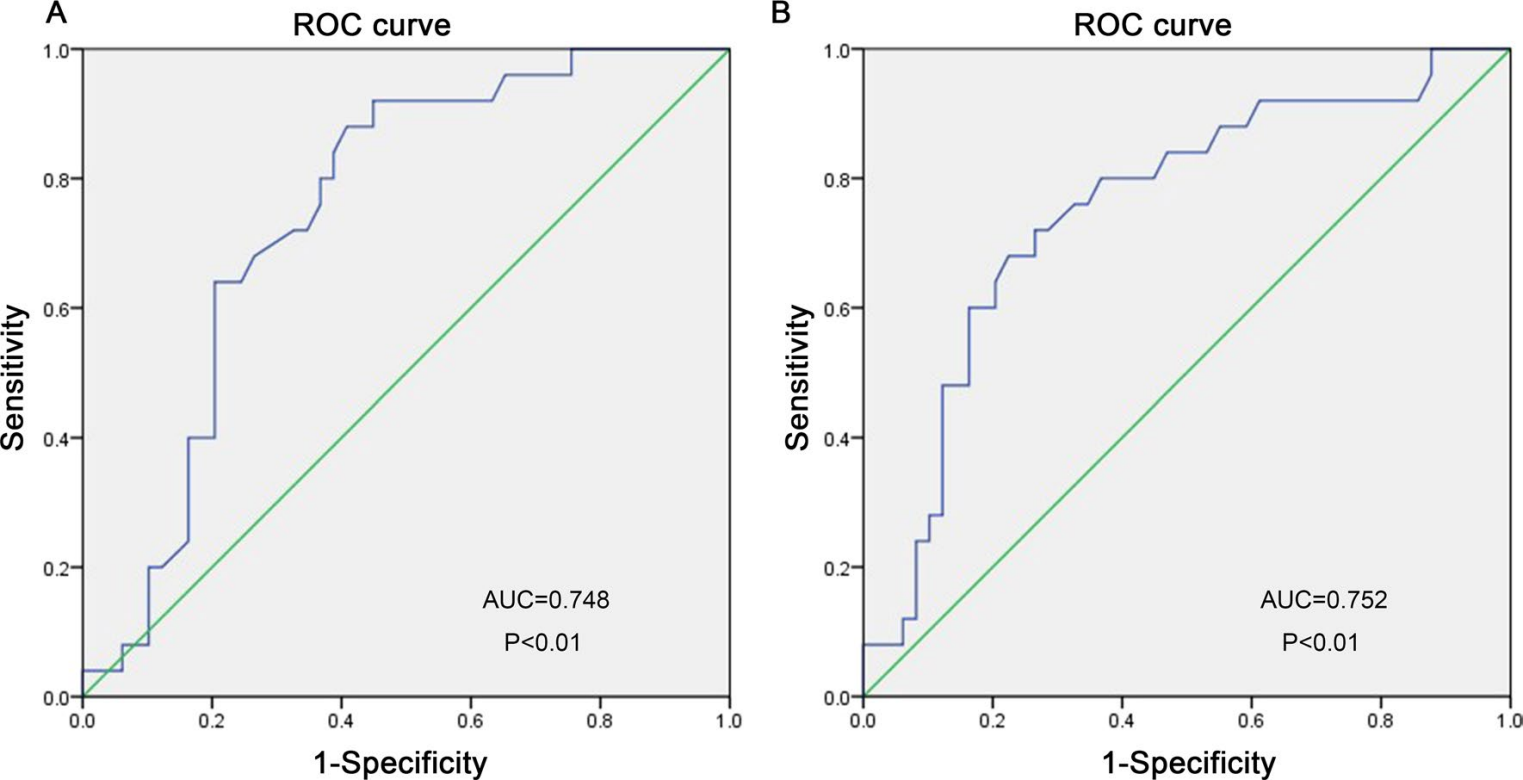
Receiver operator characteristic curves of RV/LV ratio (**A**) and WBC count **B** according to adverse outcome in intermediate-risk APE patients

Next, a binary logistic regression analysis was carried out with all patients to determine the independent factors to predict in-hospital adverse outcomes. According to the univariate analysis, a SaO_2_ < 90%, RV/LV ratio, Syncope, NTW in V1–V3, intermediate high-risk, BNP, and WBC count were considered potential independent predictors of adverse outcomes among APE patients. After the multivariate logistic regression, a SaO_2_ < 90%, (OR 5.343, 95% CI 1.241–23.008; *p* = 0.024), RV/LV ratio (OR 7.429, 95% CI 1.145–48.209; *p* = 0.036), Syncope (OR 12.309, 95% CI 1.702–89.032; *p* = 0.013), NTW in V1–V3 (OR 5.617, 95% CI 1.228–25.683; *p* = 0.026), and WBC count (OR 1.212, 95% CI 1.035–1.419; *p* = 0.017) remained as independent predictors of in-hospital adverse outcomes (Table 2). Consistently, the predictive value of the RV/LV ratio was confirmed by its ROC curve (AUC = 0.748, *p* < 0.01; Fig. 1A) and calibration (Hosmer–Lemeshow goodness of fit test, *p* = 0.070). An RV/LV ratio > 1.165 was predictive for adverse outcomes with sensitivity and specificity of 88.00 and 59.20%, respectively. The predictive value of WBC counts was also confirmed by its ROC curve (AUC = 0.752, *p* < 0.01; Fig. 1B) and calibration (Hosmer–Lemeshow goodness of fit test, *p* = 0.251). A WBC count > 9.05 was predictive for adverse outcomes with sensitivity and specificity of 68.00 and 73.50%, respectively.

## Discussion

In this retrospective study, we identified the differences in the clinical data and electrocardiography between control and adverse outcome intermediate-risk APE patients. We showed that the NTW in V1-V3, Syncope, and a SaO_2_ < 90% were more common in patients with adverse outcomes. Increased BNP, WBC count, and RV/LV ratio were also observed in the adverse outcome group compared to controls. The regression analysis showed that a SaO_2_ < 90%, RV/LV ratio, Syncope, NTW in V1-V3, and WBC count were independent predictors of in-hospital adverse outcomes among the APE patients enrolled.

The integration of independent predictors, such as echocardiographic RV dysfunction or CT, blood biomarkers, as well as clinical prediction scores, has been previously used to obtain PE risk-stratification criteria [1]. Nevertheless, a SaO_2_ < 90%, RV/LV ratio, Syncope, NTW in V1–V3, WBC count, and ECG findings have not been previously incorporated in the guidelines of PE risk-stratification criteria. The PESI and simplified PESI scores are the most frequent and validated scores used to predict the 30-days mortality of PE patients [2]. However, besides mortality, short-term adverse outcomes can not be predicted by these indicators. Hence, PE patients need additional measures to predict the incidence of adverse outcomes besides mortality. Intermediate-risk PE patients are more likely to deteriorate and would benefit from more intensive monitoring, as well as more aggressive therapeutic approaches, including thrombolysis and/or mechanical thrombectomy [4, 12, 13].

Subramanian et al. [14] have found that hypoxia is an independent predictor of adverse outcomes in PE patients, similar to our current results. Among the possible mechanisms of arterial hypoxia in APE patients, previous studies have considered that impaired oxygen transfer is caused by the mismatch of perfusion and ventilation. Other mechanisms, such as diffusion impairment, low cardiac output (as a result of RV dysfunction), as well as right-to-left shunt, can also be involved [15].

In PE patients, RV dysfunction is indicated by ECG findings, including the RBBB, S1Q3T3, and NTW in V1–V3. For example, Choi and Park have shown that the NTW in the precordial leads was the strongest independent predictor of right ventricular dysfunction in APE patients [16]. However, the pathophysiological mechanisms of the NTW in V1–V3 of APE patients remain unclear. This can be mainly explained by acute cor pulmonale due to RV dilatation followed by rapid RV pressure overload, besides RV dysfunction inducing the NTW, impairing myocardial perfusion, as well as reducing the left ventricle preload. Moreover, cellular ischemia caused by chemical mediators, including histamine and catecholamine, is also associated with NTW development [8]. In recent studies, the RBBB, S1Q3T3, and NTW in V1–V3 are frequently observed in adverse outcome groups compared to controls, regardless of the APE risk-stratification criteria. Since these indicators are surrogate markers for short-term prognosis, they have been used to predict adverse outcomes [2]. In the present study, the NTW in V1–V3 was an independent predictor of in-hospital adverse outcomes among intermediate-risk APE patients. However, the S1Q3T3 and RBBB were not significantly more frequent in the adverse outcome group than in controls.

Recently, the WBC count was considered an independent predictor for hospital readmission and short-term mortality of APE patients [17]. Consistent with previous reports, we also found that the WBC count can be used as an independent predictor for short-term adverse outcomes. The ROC curve (AUC = 0.752, *p* < 0.01) analysis showed that the WBC count has a high accuracy to assess adverse outcomes with a cut-off value of 9.05 in intermediate-risk APE patients. Right heart dysfunction is a known predictive factor for adverse prognosis and might be indicated by elevated WBC counts in APE patients. The correlations between the levels of factors VIII and VII, as well as fibrinogen and WBC count, have been previously observed. Therefore, elevated WBC counts can be used as a marker of hypercoagulability that might lead to worse prognoses [18, 19].

Here, significantly more prominent parameters that indicate RV dilatation and dysfunction were observed in the population with adverse outcomes compared to controls. The ROC curve (AUC = 0.748, *p* < 0.01) analysis showed that the RV/LV ratio has a high accuracy to assess adverse outcomes with a cut-off value of 1.165 in intermediate-risk APE patients. The RV enlargement, characterized by an RV/LV ratio ≥ 0.9, has been previously reported as an independent predictor of adverse in-hospital outcomes, both in the overall PE population and in hemodynamically stable patients [10]. Moreover, a meta-analysis, including 49 studies and more than 13,000 PE patients, has found positive correlations between a five-fold risk of PE-related mortality and/or 2.5-fold increased risk for all-cause mortality and increased RV/LV ratio ≥ 1.0 on CT [20]. In the present study, the RV/LV ratio was measured by the short axial views rather than the four-chamber reconstruction. A previous study has shown that the RV/LV ratio obtained from four-chamber views is superior to the axial views for identifying high-risk PE patients [21]. However, compared to short axial views, this method is more time-consuming and requires specific software tools, which is disadvantageous in emergencies.

Although the detailed mechanism of syncopes in APE patients is not completely understood, it is considered a concerning feature in these patients. Neurogenic syncopes and associated dysrhythmias derived from Bezold–Jarisch type vasovagal reflexes, as well as acute right ventricular failure, are considered the main mechanisms to explain PE-related syncopes. However, perfusion or ventilation abnormalities caused by hypoxemia might be significantly involved in the development of syncopes. Due to transient depressions in cardiac output, main pulmonary or lobar artery obstructions can also be associated with syncope [22, 23]. In intermediate-risk PE patients with right ventricular involvement, the presence of syncopes is associated with a more complicated in-hospital course [4]. Similar to previous reports, we demonstrated that intermediate-risk APE patients with syncope have a higher risk of clinical deterioration during hospitalization compared to patients without syncope. Although syncope has been suggested as a marker for adverse outcomes in these patients, data remain scarce. Finally, intermediate-risk PE patients are a heterogeneous group.

### Study limitations

Our current study was limited by its retrospective design. Hence, selection bias was inevitable, and can only be addressed as hypothesis-generating. Further prospective randomized studies are required to evaluate whether more aggressive therapeutic approaches in APE patients are warranted. Additionally, only a small number of patients were enrolled at a single center. Thus, our current results need to be validated on a larger, multi-center cohort. Moreover, Gülay Gök et al. [24] have found that heart rate and blood urea nitrogen are independent predictors of in-hospital mortality, and the presence of low albumin levels, dementia, and congestive heart failure are associated with higher 30-day mortality. Öz A and Hayıroğlu Mİ et al. [25, 26] have found that the levels of plasma osmolality and prognostic Nutritional Index can have a predictive value for in-hospital mortality of APE patients. Herein, we selected patients with intermediate-risk PE, and only the changes in their conditions during hospitalization were observed. Due to the small number of deaths in the sample, no clinical data affecting the 30-day mortality were detected. Moreover, Cagdas and Rencuzogulları et al. [27, 28] have found that the QRS and S zswave variations can be useful electrocardiographic signs for the diagnosis of APE. However, these indicators were not included in the current study. Finally, whether QRS and S wave variations are associated with the prognosis of PE remains unknown and should be explored in future studies.

## Conclusions

Although anticoagulation therapy is sufficient for most intermediate-risk PE patients, some patients can present a poorer prognosis in the acute phase, thereby requiring advanced and reperfusion therapies (systemic fibrinolytic agents or thrombectomy) compared to their low-risk PE counterparts. In the present study, we showed that a SaO_2_ < 90%, RV/LV ratio, Syncope, NTW in V1-V3, and WBC count can independently predict adverse outcomes of hospitalized intermediate-risk APE patients. Therefore, these patients might benefit from interim intensive surveillance, including continuous monitoring and ongoing assessment to select treatment strategies beyond anticoagulation.

## Data Availability

The original data supporting the conclusions of this paper will be provided by the corresponding authors without undue reservation.
